# Testing the effect of semi-transparent spectrally selective thin film photovoltaics for agrivoltaic application: A multi-experimental and multi-specific approach

**DOI:** 10.1016/j.heliyon.2024.e26323

**Published:** 2024-02-15

**Authors:** Maurizio Zotti, Stefano Mazzoleni, Lucia V. Mercaldo, Marco Della Noce, Manuela Ferrara, Paola Delli Veneri, Marcello Diano, Serena Esposito, Fabrizio Cartenì

**Affiliations:** a Department of Agricultural Sciences, University of Naples Federico II, Via Università 100, 80055, Portici, Na, Italy; b Italian National Agency for New Technologies, Energy and Sustainable Economic Development (ENEA), Portici Research Center, Piazzale E. Fermi, 80055, Portici, Italy; c M2M Engineering Sas, Via Coroglio, 57, Science Center, 80124, Naples, Italy; d NoSelf AND BV, Robert Schumandomein, 2 Maastricht, NL-6229, ES, the Netherlands

**Keywords:** *Lactuca sativa*, *Ocinum basilicum*, *Solanum lycopersicum*, *Chlorella vulgaris*, *Arthrospira platensis*, Agrivoltaics, Building integrated photovoltaics, Spectral selectivity, Thin film photovoltaics

## Abstract

The integration of photovoltaic technologies within the agricultural framework, known as agrivoltaics, emerges as a promising and sustainable solution to meet the growing global demands for energy and food production. This innovative technology enables the simultaneous utilization of sunlight for both photovoltaics (PV) and photosynthesis. A key challenge in agrivoltaic research involves identifying technologies applicable to a wide range of plant species and diverse geographic regions. To address this challenge, we adopt a multi-experimental and multi-species approach to assess the viability of semi-transparent, spectrally selective thin-film silicon PV technology. Our findings demonstrate compatibility with crop production in controlled environments for both plants and algae. Notably, selective thin-film PV exhibits the potential to enhance crop yields and serves as a photo-protectant. We observe that plant and algal growth increases beneath the selective PV film when supplemented with appropriate diffuse light in the growth environment. Conversely, in situations where light intensity exceeds optimal levels for plant growth, the selective PV film provides a photo-protective effect. These results suggest potential supplementary benefits of employing this technology in regions characterized by excessive light irradiation, where it can contribute to healthy plant growth.

## 1 Introduction

In recent decades, the demand for sustainable solutions in agriculture has become a central focus of research activities [[1], [2], [3], [4]]. The increasing global population has led to a growing need for food, while simultaneous efforts are essential to minimize the human footprint on the planet [[5], [6], [7]]. Research activities play a pivotal role in addressing resource management policies to achieve sustainability goals [[8], [9], [10]], particularly in the context of energy production and the availability of food resources, which are among the most impactful human activities.

**Fig. 1:**
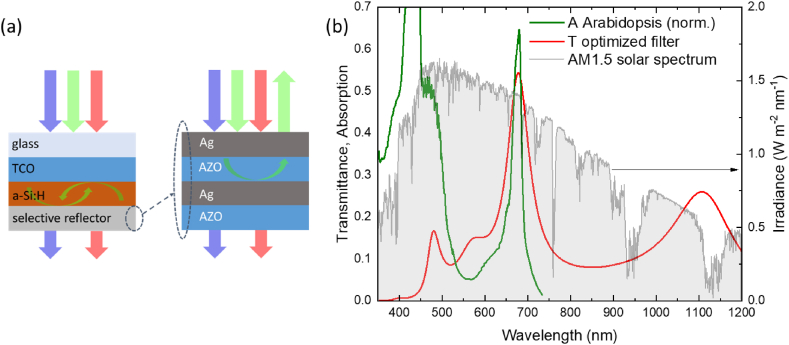
(a) Schematic representation of the spectrally selective PV filter tested in this work; (b) Simulated transmittance of the optimized layer stack (red line) designed to target the absorption spectrum of *Arabidopsis thaliana* (green line, normalized spectrum) superimposed over the AM 1.5 global solar spectrum irradiance (ASTM G-173-03 reference spectrum).

Numerous works have aimed to improve the sustainability of production processes and minimize human impact based on the agrivoltaic concept. The combination of solar radiation exploitation for both food and energy production is recognized as one of the most promising strategies towards sustainability [[11], [12], [13], [14]]. The sun provides a power density of 1000 W/m^2^ at the Earth's surface, with a spectral distribution concentrated largely in the visible to near-infrared range (Fig. 1). Plants utilize solar power for biomass or food production through photosynthesis, and it can also be harnessed to generate electricity with photovoltaic panels. Agrivoltaics represents the cooperative integration of plant growth and photovoltaic electricity production in the same area.

Initial agrivoltaic applications were designed using conventional photovoltaic (PV) modules in open fields [13]. Adequate spacing among conventional opaque PV modules is necessary to allow sufficient light to reach the plants underneath [15]. A novel alternative concept is based on the development of semi-transparent spectrally selective thin-film PV modules, eliminating the need for spacing [16]. Transparent and semi-transparent solar cells are already extensively studied for building-integrated applications [17,[18], [19], [20], [21]].

This approach is grounded in the idea that chlorophyll molecules, driving photosynthesis, only absorb specific portions of light in the blue and red parts of the spectrum. Other colors can be allocated for other functions, such as PV applications [22,23]. The goal is to achieve an integrated, complementary use of sunlight for both photovoltaics and photosynthesis by tuning the transmission of PV modules to the absorption spectrum of plants. Integrating semi-transparent PV modules into a greenhouse structure is a proposed approach in agrivoltaics for simultaneous plant cultivation and electricity generation, with the added benefit of reducing greenhouse energy demand [24]. Semi-transparent organic solar cells are primarily explored for this application, leveraging the tunable absorption characteristics of the active materials [[25], [26], [27], [28]]. Alternatively, the desired spectral allocation can be achieved with inorganic thin-film solar cells through engineered multilayer designs of the back reflecting contact [29].

In this context, we investigate plant growth under a novel implementation of the latter approach, specifically spectrally selective PV based on well-established thin film silicon PV technology [30,31]. To the best of our knowledge, most current works focus on testing the effect of a selected PV technology on single species, often in open field conditions [32], or concentrate primarily on the photovoltaic aspects [33,34]. In other words, the research effort has primarily aimed at maximizing the efficiency of PV modules rather than testing adaptability to the requirements of different crop species, limiting the technology's applicability. On the other hand, multi-specific plant production systems should be considered, as crop rotation is essential in the field to maintain adequate productivity. Moreover, many studies in the literature are based on observations in controlled conditions and do not analyze the effect of PV on plant growth when exposed to different light sources. This may mask biases due to variations in sunlight during the day, restricting applicability to geographic areas with specific daylight radiation [33,35].

To overcome the current limitations in knowledge regarding the applicability of semi-transparent PV modules, we designed a multi-experimental assessment while evaluating an implementation of such modules that is easy to transfer to production. Specifically, we focused on the following aspects.

1. Different light sources.
2. Different configurations impacting light irradiation on plants.
3. The effect on growth across different photosynthetic organisms.
4. The applicability of the selected PV technology considering the agronomic needs of different photosynthetic organisms.

## 2 Materials and methods

### 2.1 PV module design, experimental box and light measurement

We opted for mature thin-film Si technology (30,31) to design and fabricate spectrally selective PV pseudo-modules, hereafter referred to as PV films or filters due to their function as light filters for plants. Although electrically non-functional, these pseudo-modules incorporate all the essential functional layers, faithfully mimicking the transmittance of real modules. This setup facilitates an in-depth investigation into the impact on plant growth and physiology.

In contrast to conventional opaque solar cells employing a metal back-reflecting contact, both front and back electrodes in our design need to be transparent. Fig. 1A illustrates the schematic of these filters. They comprise a semiconducting core of hydrogenated amorphous silicon (a-Si:H) sandwiched between transparent electrodes using transparent conducting oxide (TCO) films. The front electrode is a single TCO layer, similar to conventional designs, while the back electrode consists of a meticulously engineered metal/TCO/metal/TCO Fabry-Perot-type reflector to achieve the desired spectral selectivity [29]. We employed Al-doped ZnO (AZO) as TCO and Ag as the metal [36]. The complete sample consists of six layers: AZO/a-Si:H/Ag/AZO/Ag/AZO, applied on the glass substrate in this order, with semi-transparent ultrathin metal layers. The intrinsic a-Si:H layer optically emulates the full semiconducting stack of electrically functioning solar cells, where this material (the light absorber) is situated between selective charge carrier collectors made of ultrathin silicon-based doped layers (p-type on one side and n-type on the opposite side) with no significant impact in optical terms.

**Fig. 2:**
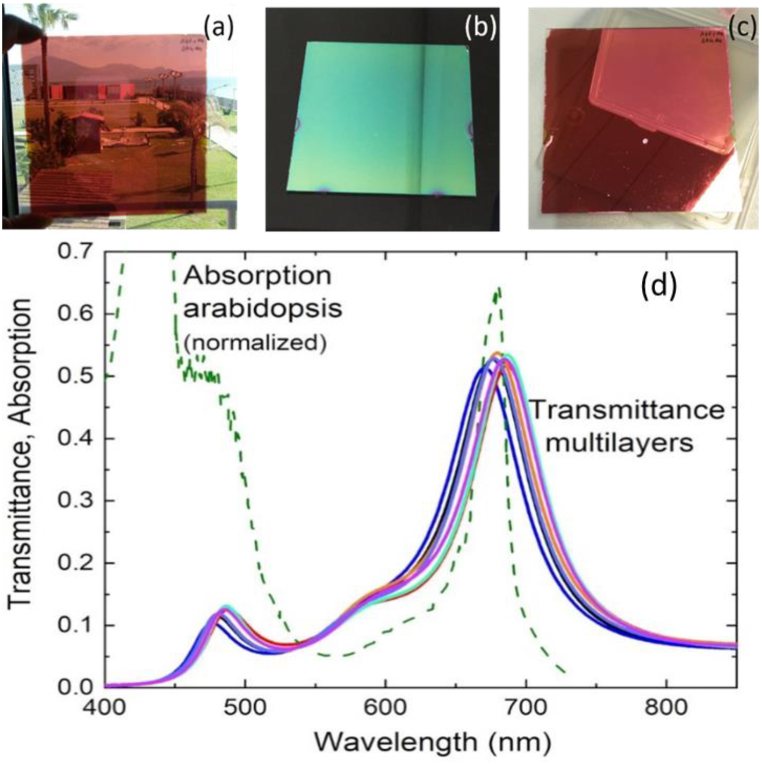
Top: photographs of one of the PV filters (10 cm side) with different background and/or orientation of the sample, showing transmission (a) and reflection color of the prototypes from the film (b) and glass (c) side, respectively. Bottom: experimental transmittance spectra of a set of nominally identical samples compared to the normalized absorption spectrum of *Arabidopsis* (d).

The entire multilayer is only approximately 0.7 μm thick. We fixed the thickness of the a-Si:H layer at 70 nm, relatively thin compared to conventional amorphous silicon solar cells, to ensure sufficient transmission, and the front AZO thickness to 180 nm. Regarding the back electrode, we selected layer thicknesses based on optical modeling of the full stack, aiming for a transmittance spectrum adjusted to the absorption spectrum of the model organism *Arabidopsis thaliana* (Fig. 1B). Simulations were conducted using the IMD software for optical modeling of multi-layer films, utilizing the experimental optical constants of all films obtained through spectroscopic ellipsometry [37,38]. Fig. 1B presents the simulated transmittance of the optimized stack, with an Ag thickness of 12 nm and AZO thicknesses of 330 nm and 100 nm, respectively, for the intermediate and final layers. Alongside a good match with the absorption spectrum of *A. thaliana*, the figure highlights an additional potentially beneficial feature of these filters for the proposed application: a substantial reduction of the infrared light transmitted to the underlying plants. Fig. 2 a - d displays photographs of a sample under natural light with its reflection and transmission color, along with the experimental transmittance spectra of a set of nominally identical samples, in good agreement with the model prediction.

In practice, we fabricated the samples on 10 × 10 cm^2^ glass substrates (Corning Eagle XG). The a-Si:H film (70 nm thick) was deposited using plasma-enhanced chemical vapor deposition (PECVD; Plasmalab800+, Oxford Instruments) at 150 °C with pure silane gas at a pressure of 300 mTorr and a power density of 36 mW/cm^2^. The AZO films, with thicknesses of 180 nm, 320 nm, and 100 nm in this order for the three layers in the stack, were deposited by RF-sputtering (multi-target MRC 643 sputtering system) from a ZnO:Al target with 2 wt% Al_2_O_3_ doping, with Ar pressure of 6 mTorr and power density of 2.2 W/cm^2^. The Ag films (12 nm thick) were deposited by DC-sputtering in the same multi-target system at 300 W and Ar pressure of 10 mTorr. Film transmittance was measured with a PerkinElmer λ-900 spectrophotometer. Single layers were deposited on glass using the same methods for optical characterization via spectroscopic ellipsometry (VASE, J.A. Woollam Co., Lincoln, NE, USA).

**Fig. 3:**
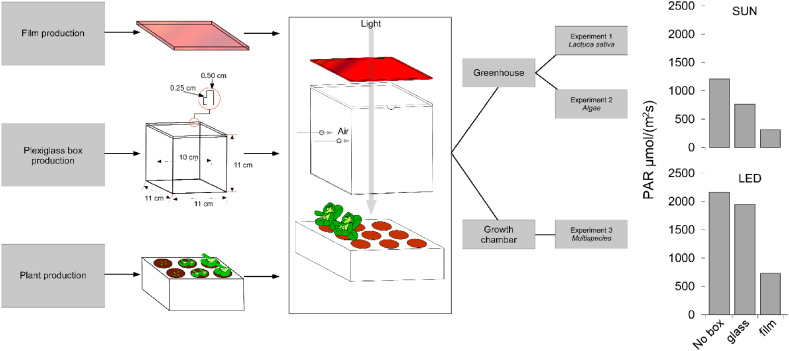
Workflow diagram of the production steps of selective films, plexiglass boxes and photosynthetic organisms and experimental setup. Common experimental units were used for experimentation in two different light conditions and with different photosynthetic organisms. Light intensity measured in PAR μmol m^−2^ s^−1^ for no box, glass and film conditions are showed in barplots.

To hold the PV glass plates above the plants, plexiglass boxes were designed and produced via laser cutting. The boxes comprised four lateral walls (0.5 cm wide) forming a cube with internal dimensions of 10 × 10 × 10 cm^3^. Each wall had a small internal step at the top (0.25 cm depth) to hold the 10 × 10 cm^2^ glasses (see Fig. 3). Additionally, two holes with a 0.5 cm diameter were created on each side wall to facilitate air circulation.

Light flux intensity was measured as the photosynthetic photon flux density (PPFD) using a spectroradiometer (MSC15, Gigahertz-Optik, Turkenfeld, Germany). Initially, light intensity was measured in the environment without the experimental box (no-box). Subsequently, the experimental box was placed on the spectroradiometer, and measurements were conducted with transparent glass (glass) and using the selective film (film). All measurements were reported in photosynthetically active radiation (PAR).

### 2.2 Experiment 1– lettuce under PV film in natural sunlight

Experiment 1 aimed to assess how the PV film alters natural solar radiation and the subsequent impact of this altered light input on plant growth. The growth experiments were conducted under various conditions: without any glass cover (control) and with a glass cover with or without the PV film. To comprehensively investigate and isolate the effects of the PV film cover, two additional experimental factors were introduced: the inclination of the glass cover, which could either be flat (no-tilt) or tilted by 30°, and the transparency of the lateral walls, which were either transparent to allow the passage of diffuse light from side walls or completely opaque.

The complete experiment resulted in a full factorial design, encompassing the glass cover with or without the PV film, different inclinations of the PV film at 0° or 30° slope (Tilt), and box sidewalls with or without an opaque adhesive layer (lateral transparency). Control seedlings were allowed to grow without the application of any cover structure. The experiments took place in the historical greenhouse of the Orto Botanico di Portici, in the Department of Agricultural Sciences of the University of Naples Federico II. This greenhouse, positioned for optimal light exposure (South-west), is dedicated to the cultivation of rare crassulacean plants. Lettuce seedlings, sourced from a local producer, were used starting form a height of around 5 cm. Seedlings exhibiting variations in biometrics, such as a higher number of leaves, anomalous development of the root apparatus, or higher/lower length of the aerial part, were discarded and not included in the experiment.

Each experimental unit consisted of five seedlings placed in polystyrene cell seed trays cut to match the dimensions of the experimental box. The experimental units were then constructed by layering seed trays with plants, boxes, and selective films (see Fig. 3). All systems were placed in plastic trays filled with water and nutrient solution (refer to experiment 3 for detailed information on the solution composition) to facilitate floating and fertigation. At the conclusion of the 21-day experiment, only biometrics of aerial parts were collected, as the use of commercial seedlings led to the homogenization of the root apparatus through cell tray growth (total root apparatus weight around 13 g, including the soil used for germination). Specifically, the measured variables were.

1. Total Biomass
2. Aboveground Biomass
3. Number of leaves (excluding cotyledonary leaves)
4. Shoot length (plant height).

### 2.3 Experiment 2 – microalgae under PV glass in natural sunlight

We conducted another experiment in natural sunlight using microalgae as the photosynthetic organisms under investigation. Two commercially available microalgal species, *Arthrospira platensis* and *Chlorella vulgaris*, were selected for testing.

In this trial, our objective was to assess the impact of the PV film on algal growth, including the exploration of a potential tilt to enhance light penetration. Both algae species were cultivated in the same greenhouse utilized for Experiment 1. Each experimental unit comprised algae growing in 100 ml flasks placed inside the experimental box with fully opaque side walls (see Fig. 3). The initial inoculum was approximately 0.3 g/l for *A. platensis* and 0.2 g/l for *C. vulgaris*. The inocula were placed in 65 ml of nutrient solution, and measurements were taken at various time steps to monitor algal growth throughout the exposure period. The experiment spanned 26 days, and growth was quantified by biomass expressed in g/l. All flasks were sealed with cotton caps to prevent contamination and allow transpiration.

### 2.4 Experiment 3 – High light intensity in growth chamber

#### 2.4.1 Growth chamber and settings

Plant growth bioassays were conducted at the Department of Agricultural Sciences, University of Naples Federico II, in Portici, within a growth chamber (KBP–6395F, Termaks, Bergen, Norway). The chamber was equipped with an LED panel unit (K5 Series XL750, Kind LED, Santa Rosa, CA, USA), emitting light in the 400–700 nm wavelength range.

The LED panel, situated inside the growth chamber, ensured a uniform distribution of light (2000 ± 10 μmol m^−2^ s^−1^) across the entire shelf surface (0.4 m^2^). Despite this uniformity, polystyrene cell seed trays containing plants were rotated every 24 h to enhance irradiation uniformity from the light source. Light parameters, including photosynthetic photon flux density (PPFD) and light spectra in the chamber, were controlled using a spectroradiometer (MSC15, Gigahertz-Optik, Turkenfeld, Germany). Air temperature and relative humidity were set at 24/18 °C and 75/85%, respectively. The alternation between day and night was maintained by configuring a photoperiod of 16 h of light and 8 h of darkness.

#### 2.4.2 Plant material preparation

The species tested in the experimental assay included *Lactuca sativa* var. *canasta*, *Lycopersicon esculentum* var. *pachino*, and *Ocimum basilicum* var. *genovese*. Plant materials were obtained through seed germination in polystyrene cell seed trays with a diameter of approximately 3 cm. Seed trays were sterilized, to prevent the emergence of soil-borne pathogens, with quaternary ammonium salts and washed in distilled water three times, and then left to dry in a microbiological laminar flow hood. Simultaneously, seeds were surface-sterilized by soaking them three times in a 4% sodium hypochlorite solution for 15 min, with washing in distilled water between each phase. Sterilized seeds were dried in a laminar flow hood, followed by a one-week stratification period in the dark at 4 °C to ensure consistent germination. Two seeds per cell were then germinated in the seed trays, using a soil mixture of peat sphagnum and perlite in a 3:1 ratio, previously autoclaved at 121 °C and 3 bar for 15 min. Following germination, seedlings were thinned to one plant per cell after two days of emergence. Subsequently, plants were grown until they were used for the experiment.

#### 2.4.3 Experimental design

The experimental design aimed to assess the growth of *L. sativa* (lettuce), *L. esculentum* (tomato), and *O. basilicum* (basil) under different light conditions. Similar boxes to those used in previous experiments were employed with sidewalls covered with opaque adhesive film to eliminate lateral light contributions. These boxes, closed at the top with either bare glass or PV glass, were placed on each seed tray to create an isolated environment for plant growth. The number of seedlings per box varied according to the size of the plant species. For tomatoes, five plants with an average starting height of 7.78 cm were used, while lettuce and basil utilized nine seedlings each, with initial heights of 5.26 cm and 4.48 cm, respectively. To monitor the growth process effectively, measurements were collected at three time steps: an initial phase at the start of the experimental essay (T0), an intermediate phase after five days (T1), and a final phase after five more days (T2). The entire experiment lasted a total of 10 days.

Due to the need for destructive sampling in phases T1 and T2, two blocks for two light conditions were designed within each seed tray, resulting in a total of 12 boxes used in the experiment. Polystyrene trays were placed in aluminum trays for sub-irrigation during the experiment. In addition to irrigation water, Hoagland solutions were added, with fertilizer solutions comprising 2.0 mM nitrate, 0.25 mM sulfur, 0.20 mM phosphorous, 0.62 mM potash, 0.75 mM calcium, 0.17 mM magnesium, 0.25 mM ammonia, 20 μM iron, 9 μM manganese, 0.3 μM copper, 1.6 μM zinc, 20 μM boron, and 0.3 μM molybdenum. The electrical conductivity (EC) was maintained at 0.35 dS m^−1^, and the pH was set to 6.0.

For each time step in the experiment, the following biometric data were collected.

1. The number of true leaves (excluding cotyledonary leaves).
2. Plant height.
3. Dry and fresh root biomass.
4. Dry and fresh biomass of the stem.
5. Dry and fresh leaves biomass.
6. Dry and fresh total biomass.
7. Root biomass/Shoot biomass.
8. Leaves area.
9. Leaves length.
10. Leaves width.
11 SPAD (measurement of chlorophyll with absorbance between 650 and 950 nm). Measurement of SPAD is available only for the T2 time step.

A summary of the experimental workflow is presented in Fig. 3.

### 2.5 Data analysis

To evaluate the overall significance of the experimental conditions, multivariate analyses of variance (MANOVA) were conducted using the entire set of variables in each test. Subsequently, each dataset underwent specific statistical analyses to validate individual experimental hypotheses.

In the first set of experiments, data for Shoot biomass, Number of leaves, and Shoot length for *Lactuca sativa* were represented as a percentage of deviation compared to controls, with mean values of controls set to zero. The objective of this experiment was to assess biometric variations in *Lactuca sativa* plants growing in boxes with transparent or opaque sides, with tilted or flat covers, and below PV film or bare glass against full sun (controls with no cover). To compare sampling groups from the factorial experimental design for each variable, two-sample T-tests for grouped data were employed. Prior to the statistical test, percentage data were arctangent transformed to address heteroskedasticity issues, and normality was tested using the Shapiro-Wilk test. Significance was assigned for a p-value <0.05.

The dataset from the microalgae experiment was analyzed using repeated measure ANOVA, with cover type as the categorical predictor and algal biomass as the dependent variable for each time step. Analyses were conducted separately for each algal species tested in the experiment. Post hoc tests of pairwise comparisons between cover types at each time step were performed using the Tukey HSD test. Similar to the first experiment, two separate analyses were conducted for each algal species. The significance level was set at a p-value <0.05, and data were log-transformed to mitigate bias from heteroskedasticity and heterogeneous variance.

In the multispecies experiment (Experiment 3), biometric variables were analyzed for significant changes based on the presence of bare glass and PV film, applying post-hoc Dunnett tests. Comparison between time-step data was not considered due to dependency within sampling groups in consecutive time steps. The Shapiro-Wilk test was applied to assess normality in data distribution, and no transformation was deemed necessary. Significance was set at values of p below 0.05.

All data analyses were performed using Statistica 10 software (TULSA, USA).

## 3 Results

### 3.1 Light measurement in greenhouse and growth chamber

As shown in Fig. 3, light intensity values varied dramatically based on experimental settings and treatments. In the greenhouse environment, the light radiation measured at the beginning of experiments 1 and 2 (i.e. on March 19th, 2021) reached PAR values around 1212 μmol m^−2^ s^−1^. In treatments where boxes were placed with transparent glass, PAR decreased to average values of 768 μmol m^−2^ s^−1^, while for boxes with selective film, average values of 308 μmol m^−2^ s^−1^ were recorded. In the growth chamber with LED light, light measurements followed similar patterns. In the experimental environment, PAR reached 2187 μmol m^−2^ s^−1^, while the presence of a box with a transparent glass cover reduced PAR to 1912 μmol m^−2^ s^−1^. Inside the experimental box with a selective film cover, PAR was further reduced to 857 μmol m^−2^ s^−1^.

### 3.2 Experiment 1 – lettuce under PV glass in natural sunlight

**Fig. 4:**
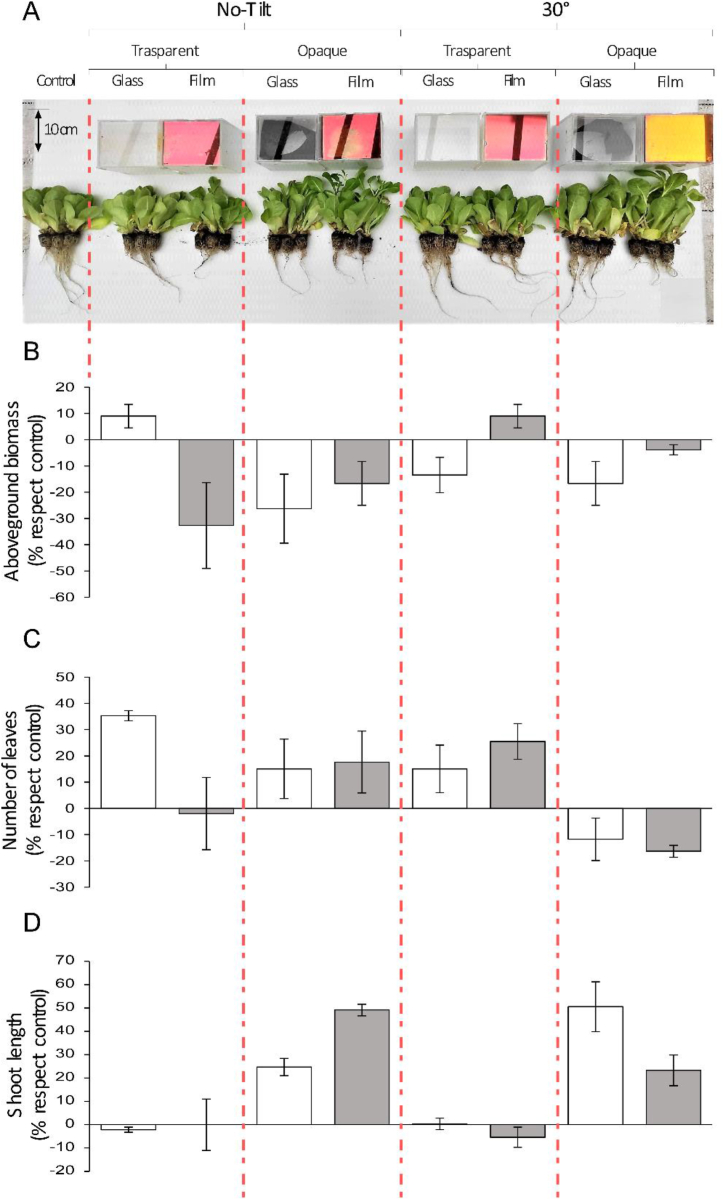
Results of experiment 1 with lettuce plants grown under natural light conditions. Pictures (A) show the experimental conditions and lettuce plants at the end of the experiment. Subsequent plots report the results for the following measured variables: (B) aboveground biomass; (C) number of leaves; and (D) shoot length. Bars indicate standard deviation. Data are expressed as percentage compared to the control group. Statistically significant differences of the three variables analyzed with respect to control are showed in Table S1.

In this experiment, we observed the performance of lettuce grown under natural sunlight with different factorial configurations of the cover (Fig. 4A). The boxes containing the plants had either transparent or opaque side walls and were covered with either bare glass or a PV selective film. Control plants were grown without boxes and covers. Results of the non-directional test of significance (ANOVA) showed that among experimental factors, box transparency was the most important factor in regulating plant growth and morphology (F = 347.890; p-value<0.001). Light modulated or not by the PV films was a less predictive factor explaining the variability of data (F = 65.184; p-value = 0.002). The inclination of the cover glass plates showed no considerable effect on the experimental data (F = 23.321; p-value = 0.101) (Table S1).

In detail, for total aboveground biomass (Fig. 4B), all treatments showed reduced growth compared to the control, with only two exceptions: (i) boxes with transparent walls and flat bare glass and, interestingly, (ii) boxes with transparent walls and tilted PV film. Both treatments increased aboveground biomass by around 10% compared to control plants. Regarding the number of leaves, all treatments showed higher relative values compared to controls except for plants grown in opaque boxes with tilted covers (Fig. 4C). Finally, the shoot length showed high responses to box opacity, with plants presenting considerable shoot elongation in the case of limited light exposure (opaque boxes in Fig. 4C). Statistical comparisons of experimental groups (Table S2) confirm the statistical significance of the differences compared to controls presented in Fig. 4.

### 3.3 Experiment 2 – microalgae under PV film in natural sunlight

**Fig. 5:**
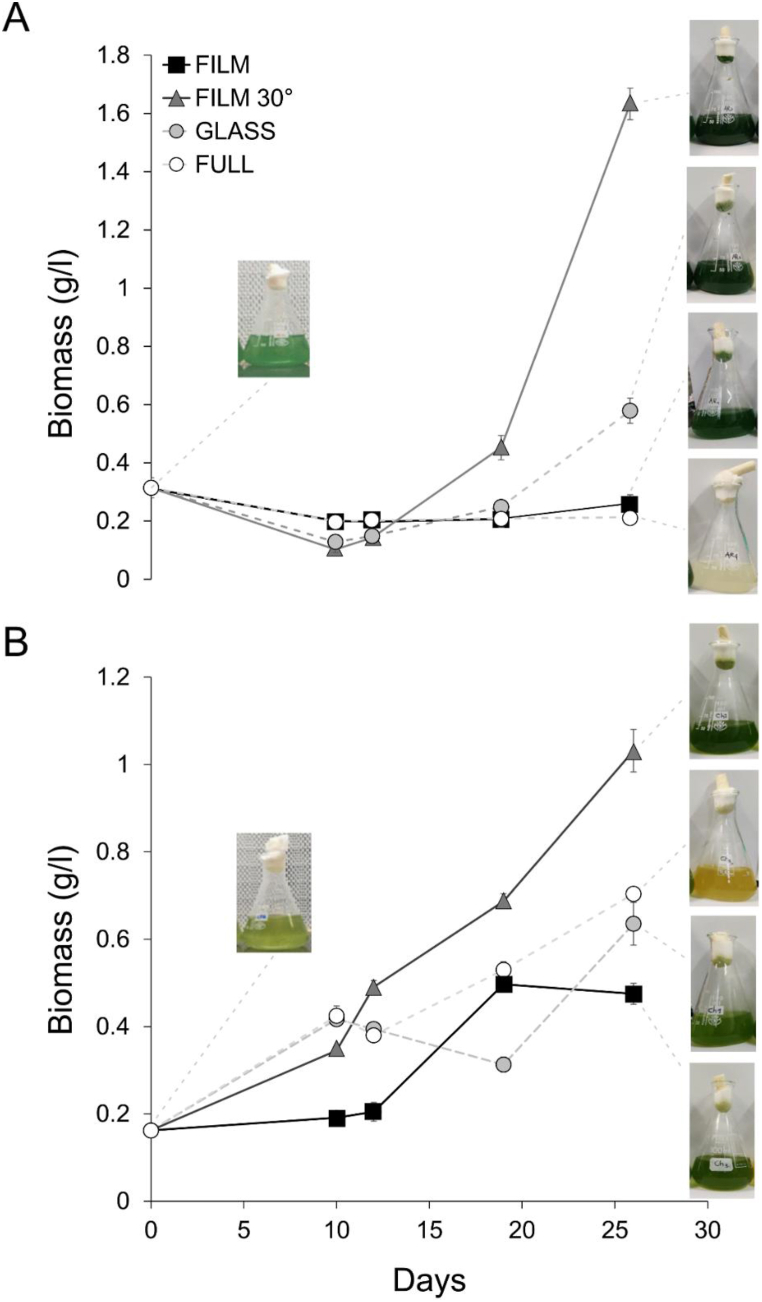
Results of the experiments with the microalgae *A. platensis* (A) and *C. vulgaris* (B) after 27 days of exposition to natural light. Boxes around the cultures were covered with horizontal glass with selective film (film); 30° titled glass with selective film (film 30°); transparent glass (glass); and full sunlight without any cover (full). Inset pictures show the color of the algal colonies at the beginning and at the end of each experimental treatment.

In this experimental trial, we observed the performance of two commercially relevant microalgal species, *A. platensis* and *C. vulgaris*, grown under natural sunlight in opaque boxes covered with either bare glass or a PV selective film. The results, presented in Fig. 5, show that algal growth is possible below selective films, but only when the PV film had a 30° inclination. In detail, *A. platensis* showed strikingly higher production below tilted selective films, while almost no growth was observed in the other conditions (Fig. 5A). Cultures grown in the control condition showed marked discoloration associated to the colony death. Cultures grown under flat selective films showed no growth but no collapse of the colony, while cultures under bare glass resulted in viable colonies with very limited biomass production (Fig. 5A). In the same conditions, cultures of *C. vulgaris* showed similar trends compared to *A. platensis* (Fig. 5B). Indeed, the microalgal population was able to grow even in full sunlight, although the first signs of discoloration due to stress became apparent at the end of the experiment. Like *A. platensis*, the growth of *C. vulgaris* under the selective film with a 30° inclination was confirmed to be the best treatment that significantly affected the biomass production of the microalga (Fig. 5B). Results of the statistical analyses for this experiment are presented in Table S3 and Table S4.

### 3.4 **Experiment 3 – high light** intensity **in growth chamber**

**Fig. 6:**
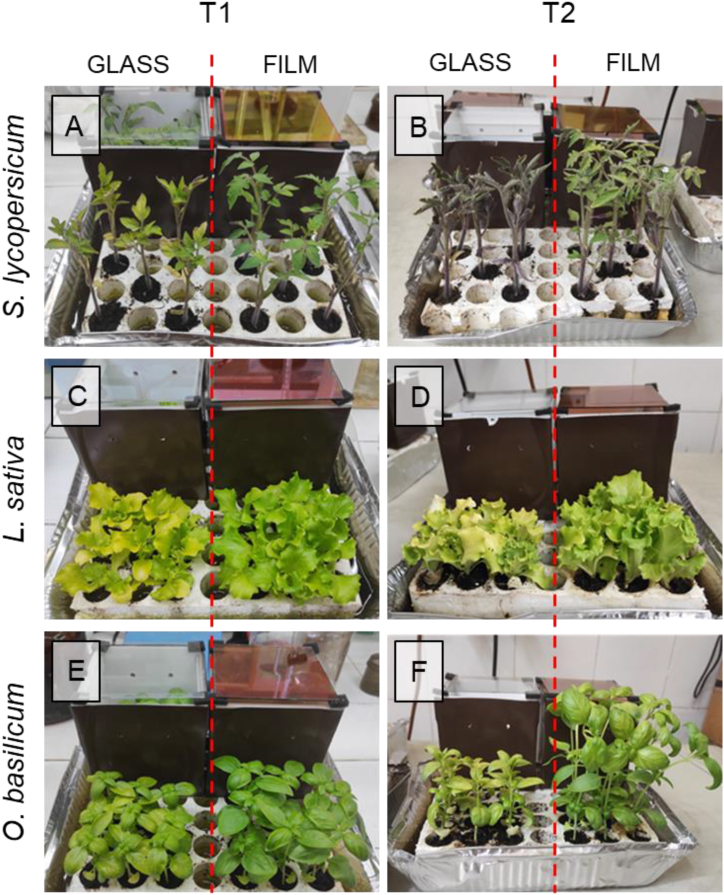
Photographs showing the plants of A-B tomato (*S. lycopersicum*), C-D lettuce (*L. sativa*) and E-F basil (*O. basilcum*) exposed to high light conditions in growth chamber after five (T1) and ten (T2) days from the beginning of experiment 3. Plants were grown within an opaque box covered with either a transparent glass (left side) or a glass with semi-transparent spectrally selective thin films (right side). Results of statistical differences in plant biometrics are shown in Table S2.

This experiment was conducted in a growth chamber, involving three commercially relevant plants—tomato, lettuce, and basil. The plants were exposed to high-intensity irradiation (2187 μmol m^−2^ s^−1^) to assess the potential positive effects of spectrally selective thin films as a protectant against excessive light stress. Upon initial observation (Fig. 6a - f), a generally positive impact was noted for plants grown beneath the PV selective film (857 μmol m^−2^ s^−1^) compared to those grown under bare glass (1912 μmol m^−2^ s^−1^). This was further confirmed by the noticeable increase in specific biometrics, as depicted in Figs. S1, S2, and S3. Multivariate analysis of variance (MANOVA) revealed that plants exhibited distinct responses over time and under different light conditions (Table S5). In the case of basil, the selective film's filtering action significantly and dramatically influenced plant growth (F = 41.567; p-value <0.001) compared to the impact of time (F = 7.416; p-value <0.001). For tomato plants, both time and light played roles in affecting plant growth, with time being the more influential factor (F = 17.270; p-value <0.001) and light as a secondary factor (F = 8.239; p-value = 0.003). Conversely, lettuce did not show any significant effect of light, but a noticeable change in growth over time was evident (F = 6.704; p-value <0.001) (Table S5). Consistent with the multivariate test of significance, the Dunnet test (Table S6) yielded similar results. In the case of basil, all variables exhibited significant changes in plants growing under different light regimes, with the most substantial effect observed after 10 days (T2 time step). Tomato plants also displayed higher changes after 10 days, with significant variations in parameters such as the number of leaves, shoot length, shoot biomass (both fresh and dry), and the root-to-shoot (R/S) ratio. For lettuce, minimal changes were observed, except for the R/S ratio after 5 days (T1 time step in Table S6). Additionally, lettuce and basil exhibited higher SPAD levels when growing under bare glass compared to PV film (Fig. S4).

## 4 Discussion

Adding semi-transparent, spectrally selective PV to a greenhouse has the potential to facilitate simultaneous crop production and electricity generation, making optimal use of the roof space while also reducing the greenhouse's energy demand. However, the impact of the PV film on plants needs thorough investigation and clarification. In this study, we conducted a multi-species examination of the effects of spectrally selective thin-film Si PV on photosynthetic organisms. The thin-film Si PV technology employed is a well-established option for cost-effective, large-area photovoltaic applications, with advantages such as the abundance and non-toxicity of silicon, high versatility, and the availability of turn-key large-area production lines [31]. The spectrally selective version of thin-film Si PV modules proposed in this study consists of ultrathin layers of inorganic materials (a-Si:H, AZO, Al) on glass, with optimized thicknesses achieved through optical modeling. Specifically, the implementation of an engineered multilayer back reflecting contact enables tailoring the transmission spectrum of the module to the portion of light essential for plant growth, namely the blue and red spectra [26]. Despite the PV module using a limited portion of the solar spectrum for electricity production, resulting in reduced power conversion efficiency compared to conventional opaque modules, this reduction is offset by the added value of potential simultaneous crop production.

In this work, we conducted three separate groups of experiments to assess the impact of the proposed PV film on the growth of photosynthetic organisms under different light sources. The first two experiments were carried out in a greenhouse with natural light conditions. In the first experiment, we observed a general reduction in the growth of *L. sativa* (aboveground biomass) with the notable exception of plants grown under tilted selective PV films in transparent boxes (Fig. 4B). However, achieving good levels of leaf biomass does not necessarily guarantee market suitability. The results of the first experiment revealed signs of light deficiencies (Fig. 4D), such as internode elongation [39,40], indicating potential marketability issues for crops like lettuce that are sold in a controlled height form [41,42]. This concern may not apply to other species.

Regarding the aboveground biomass of lettuce, in transparent boxes with transparent glass cover and no tilt configuration, plant growth increased approximately 10% compared to controls. Similar results were observed in plants growing below tilted selective PV films in transparent boxes. In the first case, the increased growth could be attributed to the combination of optimal irradiation (transparent box plus transparent glass), and the protective effect of the box limiting evapotranspiration. Plants experiencing lower water loss through evapotranspiration are less prone to hydric stress, allowing them to allocate energy to biomass growth and CO_2_ fixation [43,44]. In the second case, involving a transparent box and tilted selective PV films, it can be hypothesized that the combination of diffuse light from the side and a tilted cover allows optimal light penetration into the experimental box. This supplementation is indeed particularly crucial given the signs of light deficiency observed in the opaque box and non-tilted conditions evidenced by the excessive shoots’ elongation (Fig. 4D). Furthermore, it would be worthwhile to delve deeper into the role of the internal side of the PV selective film in reflecting and redirecting diffuse light from the side wall. This aspect is known to favor plant growth more effectively than a direct light source, as it enables a more efficient utilization of light radiation by plants, as demonstrated in previous studies [[45], [46], [47]]. For both configurations, further experimentation with detailed monitoring of microenvironmental conditions within the boxes could enhance our understanding of the processes at play.

Results from the microalgae experiments (Fig. 5) demonstrated a clear growth advantage below the selective films, especially when exposed to diffuse light (i.e. PV film tilted at 30°). It is well-established that an excess of light can have detrimental effects on microalgal populations, as the absorbed light cannot be efficiently utilized in the CO_2_ assimilation pathway, ultimately leading to cell death [[48], [49], [50], [51]]. In fact, the selected algae belong to a category of organisms with low light requirements, with optimal conditions ranging from 70 to 140 μmol m^−2^ s^−1^ for *Chlorella* [52] to 120–200 μmol m^−2^ s^−1^ for *Arthrospira* [53]. Considering the light passing through the PV selective film at the time of the experiments, it is reasonable to expect that growth is favored under the tilted PV film where a peak PAR of 308 μmol m^−2^ s^−1^ was recorded. Lower levels of growth were indeed observed in the experimental units exposed to far higher light levels under either no glass or transparent glass cover (Fig. 5), with measured peaks of PAR of 1212 and 768 μmol m^−2^ s^−1^, respectively. Therefore, it is plausible to suggest that PV selective films act as photo-protectors, allowing continuous growth in the algal population over time. Similar to the observations in Experiment 1 with lettuce, the presence of a tilted cover facilitates the passage of further diffuse light, which can be utilized for CO_2_ fixation. Notably, the presence of diffuse light is beneficial for both algal growth and greenhouse crops, as it allows for a more even distribution and utilization of light radiation by photosynthetic organisms [[54], [55], [56]]. The application of spectrally selective PV films appears particularly suitable for microalgal species requiring protection from excessive sunlight, where the modules can harvest surplus energy for electricity production.

The initial two experiments provided valuable insights into plant behavior under natural, fluctuating light conditions. To complement these findings, we conducted an additional experiment with high irradiation levels using artificial LED light sources in a growth chamber, focusing on the potential application of the system as a photoprotective device. Notably, we observed species-specific variations in plant responses, emphasizing adaptability as the primary factor influencing a plant's suitability for integration into agrivoltaic frameworks [[57], [58], [59], [60]]. Overall, plant growth was consistently higher under the PV film compared to bare glass (Fig. 6 a-f). Excessive light, in this context, led to a shift in plant development toward root structures at the expense of aerial parts under transparent glass (Figs. S1–S3), resulting in fewer photosynthetic structures [[60], [61], [62]]. Such observations can be attributed to the stress caused by excessive light [60,63]. The enhanced growth observed under the selective PV film can be attributed to its protective role, shielding plants from harmful light fluxes [64,65]. In this experiment, a high light flux of 2187 μmol m^−2^ s^−1^ was applied, which is a reasonable value for southern regions, such as the Mediterranean area and near the equator [66,67].

In summary, our study shows how the investigated selective PV film can act as a photoprotective device for various crops with simultaneous electricity generation. Despite Experiment 3 considering a limited wavelength range compared to sunlight, it is noteworthy that the proposed PV technology automatically shields plants from damaging infrared radiation, thanks to a transmission spectrum that blocks most high-wavelength light (Fig. 1, Fig. 2). While light is essential for plant growth, protection from excessive light is equally crucial, as it impacts energy investment, leading to physiological and growth imbalances [68], increased sensitivity to pathogens [69], and alterations in the quality and quantity of crops [70]. In many regions worldwide, crop production is constrained by excessive irradiation during certain periods of the year, necessitating costly measures for crop protection, environmental conditioning, and temperature control [71,72]. For instance, in the Almeria's sea of greenhouses in Spain, or across the Sele plain in Southern Italy, the majority of greenhouse tunnels employ shaded plastic covers to prevent sun damage. In certain instances, the seasonal adjustments necessitate a considerable amount of energy to uphold optimal growth conditions for crops [73]. Spectrally selective PV could serve a dual purpose by controlling light irradiation on crops and producing energy to offset climatization costs. Although the use of plastic films remains the current cost-effective solution for crop protection in agriculture, agrivoltaic systems, with further testing, could potentially offer sustainable and durable solutions with added value.

Lastly, additional assessments of the role of selective PV films could explore temperature profiles and transmission spectra regulation. The shading effect provided by PV films may mitigate excessive temperatures within experimental environments, creating more favorable conditions for plant growth. When temperatures surpass physiological limits of ∼40 °C, photosynthesis and metabolic processes are inhibited [74]. Assessing the effectiveness of employing selective PV films in greenhouses for temperature regulation is crucial, especially considering the challenges in agricultural production associated with climate changes and global warming [[75], [76], [77]]. Furthermore, there is a need to undertake more detailed experimental trials to fine-tune the modulation of light passing through selective PV films. While our PV selective film design was based on the absorption spectrum of *A. thaliana*, a model species in plant science, the PV selective film layers' transmittance could be optimized encompassing a wide range of plant crops.

## 5 Conclusions

In this study, we present results from a multispecies, multi-experimental evaluation of the impact of spectrally selective thin-film Si PV on plant growth, serving as a proof of concept for agrivoltaic applications. This approach has the potential to mitigate the environmental impact of both agricultural and energy production activities. Overall, the findings indicate the feasibility of applying this PV technology, although further investigation on a larger scale is required.

Our observations revealed compatibility with crop production, demonstrating that spectrally selective thin-film PV has substantial potential as a photo-protectant, allowing for good plant growth and yields without compromising marketability of crops, provided the presence of diffuse light sources in the growth environment. The results suggest that the use of PV technology holds promise in regions worldwide where light irradiation is excessive during certain periods of the year.

## Founding information

This work was supported by the Italian Ministry of Environment and Energy Security in the framework of the Operating Agreement with ENEA for Research on the Electric System and under the National Recovery and Resilience Plan (NRRP), Mission 4 Component 2, Investment 1.3 - Call for tender No. 1561 of 11.10.2022 of Ministero dell’Università e della Ricerca (MUR) by the European Union -NextGenerationEU. Project code PE0000021, Concession Decree No. 1561 of 11.10.2022 adopted by Ministero dell’Università e della Ricerca (MUR), Project title “Network 4 Energy Sustainable Transition – NEST.

## Data availability statement

Data will be made available on request.

## CRediT authorship contribution statement

**Maurizio Zotti:** Writing – review & editing, Writing – original draft, Visualization, Software, Methodology, Investigation, Formal analysis, Data curation. **Stefano Mazzoleni:** Writing – review & editing, Funding acquisition, Conceptualization. **Lucia V. Mercaldo:** Writing – review & editing, Visualization, Supervision, Resources, Project administration, Methodology, Funding acquisition, Conceptualization. **Marco Della Noce:** Methodology, Investigation. **Manuela Ferrara:** Methodology, Investigation. **Paola Delli Veneri:** Writing – review & editing, Visualization, Supervision, Project administration, Methodology, Data curation. **Marcello Diano:** Methodology, Investigation. **Serena Esposito:** Investigation, Funding acquisition. **Fabrizio Cartenì:** Writing – review & editing, Writing – original draft, Visualization, Validation, Supervision, Methodology, Investigation, Conceptualization.

## Declaration of competing interest

The authors declare that they have no known competing financial interests or personal relationships that could have appeared to influence the work reported in this paper.
