# Supplementary material for: Testing the effect of semi-transparent spectrally selective thin film photovoltaics for agrivoltaic application: A multi-experimental and multi-specific approach
Source: Heliyon. 2024 Feb 15;10(4):e26323. doi: 10.1016/j.heliyon.2024.e26323 (PMC10884478; doi:10.1016/j.heliyon.2024.e26323)
Supplement: Multimedia component 1 [file mmc1.docx]

# **Title:** Testing the effect of semi-transparent spectrally selective thin film photovoltaics for agrivoltaic application: a multi-experimental and multi-specific approach.

**Authors**

Maurizio Zotti^1, *^, Stefano Mazzoleni^1^, Lucia V. Mercaldo^2^, Marco Della Noce^2^, Manuela Ferrara^2^, Paola Delli Veneri^2^ , Marcello Diano^3,4^, Serena Esposito^3^, Fabrizio Cartenì^1^.

**Affiliations**

^1 Department of Agricultural Sciences, University of Naples Federico II, via Università 100, 80055, Portici (Na), Italy.^

^2 Italian National Agency for New Technologies, Energy and Sustainable Economic Development (ENEA), Portici Research Center, Piazzale E. Fermi, 80055 Portici, Italy.^

^3 M2M Engineering sas, Via Coroglio, 57, Science Center, 80124 Naples, Italy^

^4 NoSelf AND BV, Robert Schumandomein, 2 Maastricht, NL-6229 ES, the Netherlands^

**^*^** ^Correspondence: maurizio.zotti@unina.it^

**Corresponding Author:** maurizio.zotti@unina.it

# **Supplementary Information**

#
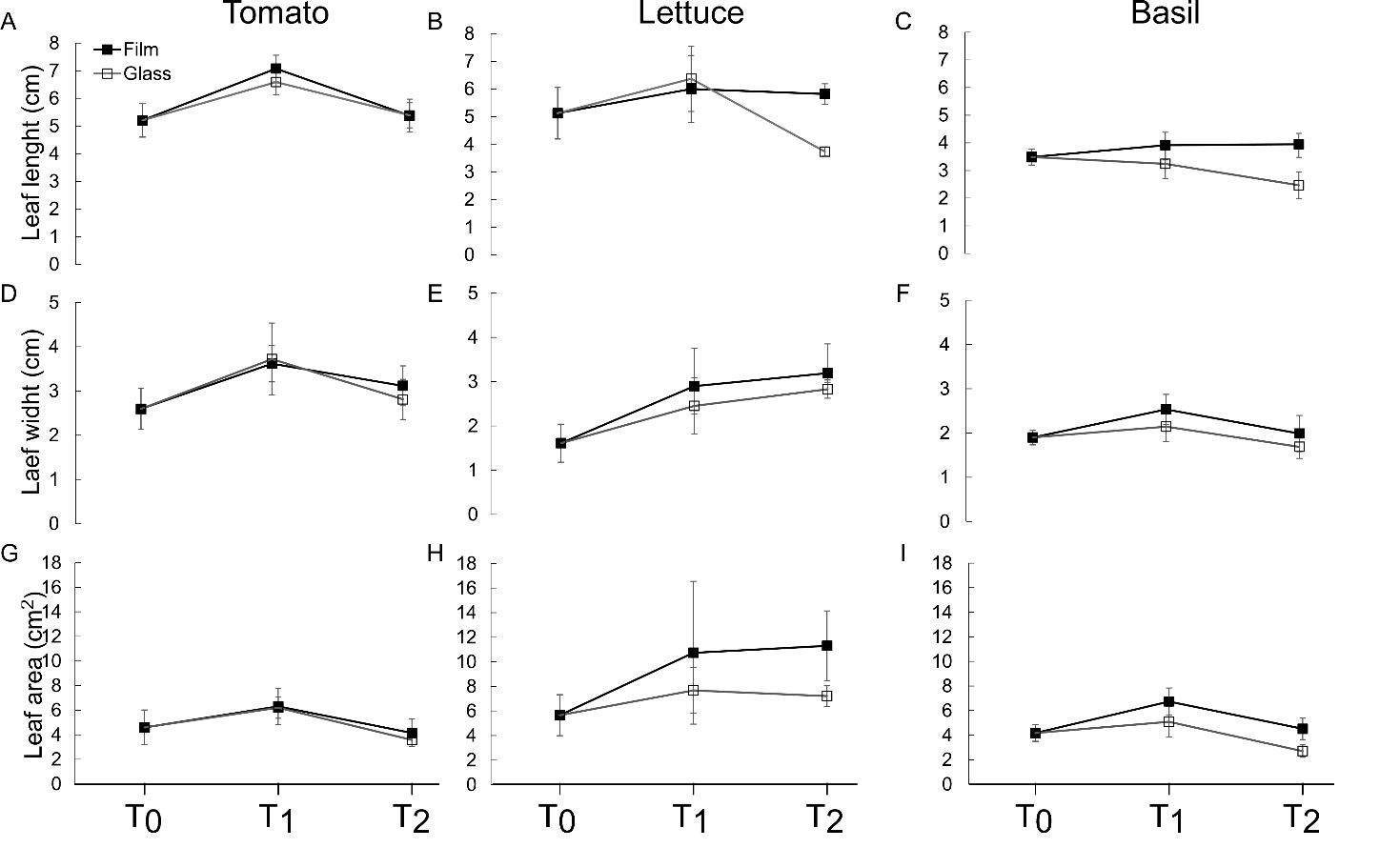


**Figure S1.** Leaf length (A, B and C), width (D, E, F) and area (G, H, I) for tomato, lettuce and basil growth in different light conditions in time (T_0_, T_1_ and T_2_ are 0, 5 and 10 days respectively). Solid squares represent leaf traits of plants grown below semi-transparent spectrally selective thin film while empty squares represent plants grown below transparent glass. Error bars represent standard deviations.


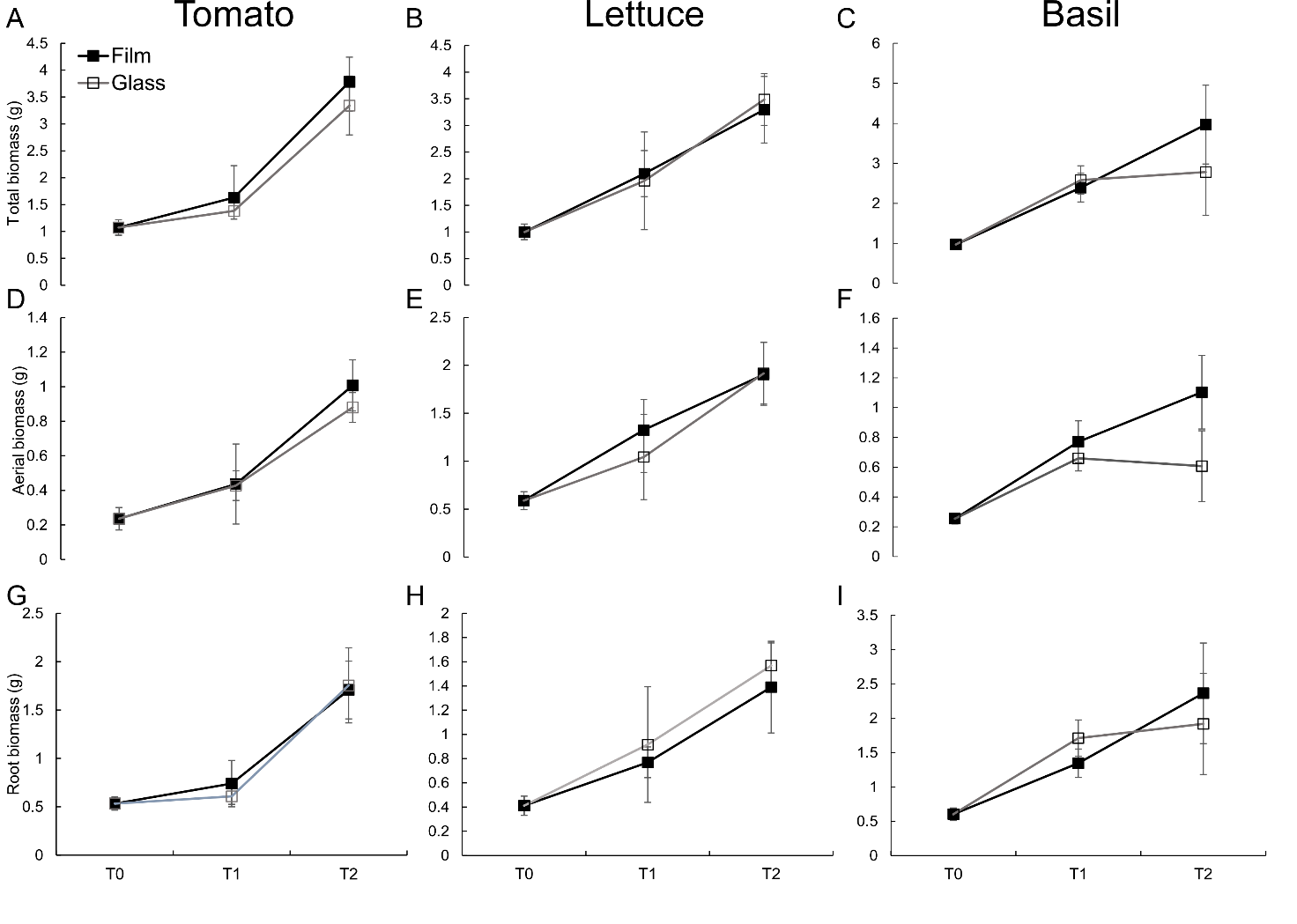


**Figure S2.** Total biomass (A, B and C), aerial biomass (D, E, F) and root biomass (G, H, I) for tomato, lettuce and basil growth in different light conditions in time (T_0_, T_1_ and T_2_ are 0, 5 and 10 days respectively). Solid squares represent leaf traits of plants grown below semi-transparent spectrally selective thin film while empty squares represent plants grown below transparent glass. Error bars represent standard deviations.


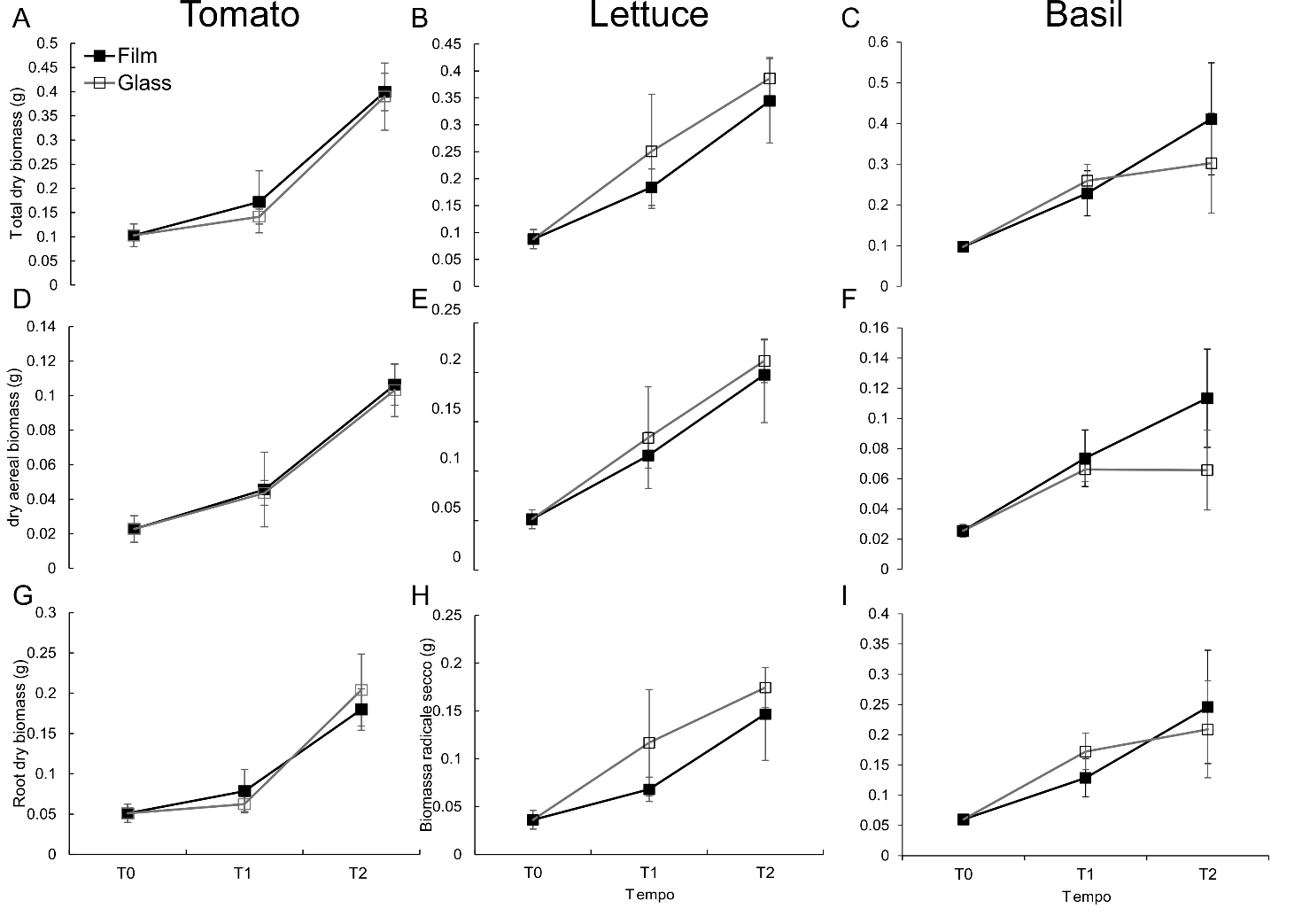


**Figure S3.** Total dry biomass (A, B and C), dry aerial biomass (D, E, F) and root dry biomass (G, H, I) for tomato, lettuce and basil growth in different light conditions in time (T_0_, T_1_ and T_2_ are 0, 5 and 10 days respectively). Solid squares represent leaf traits of plants grown below semi-transparent spectrally selective thin film while empty squares represent plants grown below transparent glass. Error bars represent standard deviations.


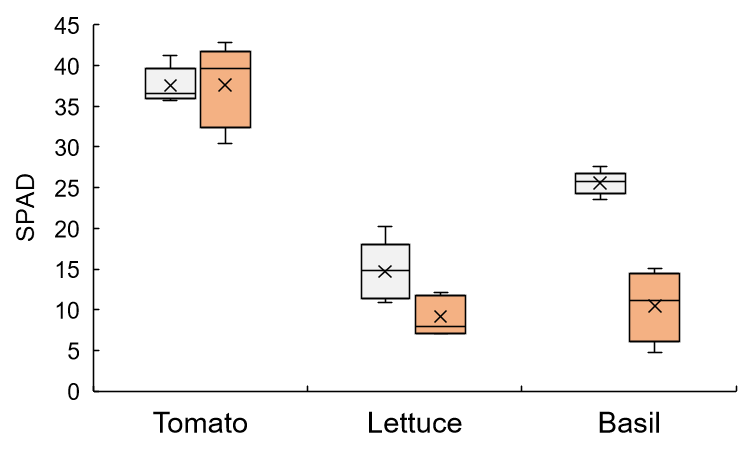


**Figure S4**. Scatterplot diagram of spad measured from plants grown under transparent glass (light grey) and semi-transparent spectrally selective thin film (orange) after 10 days in growth chamber.

**Table S1.** Results of MANOVA (Multivariate Analysis Of Variance) on lettuce plants growth in presence or absence of selective film (Light), with inclined light filters (Slope) and with or without transparent boxes (Box transparency).

|  | F | p |
| --- | --- | --- |
| Light | 65,184 | ***0,002*** |
| Slope | 23,321 | 0,101 |
| Box transparency | 347,890 | ***0,000*** |
| Light*Slope | 0,4538 | 0,717 |
| Light*Box transparency | 45,494 | ***0,012*** |
| Slope*Box transparency | 30,144 | 0,051 |
| Light*Slope*Box transparency | 11,727 | 0,342 |

**Table S2.** Results of T-test on shoot biomass, number of leaves and shoot length variables for Lactuca sativa grown with and without spectrally selective thin films in transparent and opaque boxes and different tilt of the cover. Significance is assigned for p-value below 0.05. Significant values are shown in bold.

| Inclination | Box type | Cover | Stats | Shoot biomass | Number of Leaves | Shoot length |
| --- | --- | --- | --- | --- | --- | --- |
| No tilt | Transparent box | Glass | t-value | -6,248 | -12.175 | 3.730 |
|  |  |  | p | ***0.000*** | ***0.000*** | ***0.009*** |
|  |  | Film | t-value | 4.705 | 0.249 | 0.400 |
|  |  |  | p | ***0.003*** | *0.812* | *0.700* |
|  | Opaque box | Glass | t-value | 5.521 | -2.923 | -9.615 |
|  |  |  | p | ***0.001*** | ***0.02*** | ***0.000*** |
|  |  | Film | t-value | 5.077 | -2.421 | -5.962 |
|  |  |  | p | ***0.002*** | ***0.037*** | ***0.000*** |
| 30° | Transparent box | Glass | t-value | 5.210 | -3.356 | -0.243 |
|  |  |  | p | ***0.001*** | ***0.015*** | *0.810* |
|  |  | Film | t-value | -5.474 | -5.863 | 0.864 |
|  |  |  | p | ***0.001*** | ***0.001*** | *0.280* |
|  | Opaque box | Glass | t-value | 5.337 | 3.207 | -8.009 |
|  |  |  | p | ***0.001*** | ***0.018*** | ***0.000*** |
|  |  | Film | t-value | 3.769 | 5.515 | -9.794 |
|  |  |  | p | ***0.009*** | ***0.001*** | ***0.000*** |

**Table S3.** Results of univariate repeated measure ANOVA for biomass of Arthrospira and Chlorella cultured with and without spectrally selective thin films (cover type). Significant values for p< 0.05.

|  | Species | F-value | P-value |
| --- | --- | --- | --- |
| Cover type | *Arthrospira* | ***59,954*** | ***<0.001*** |
|  | *Chlorella* | ***272,679*** | ***<0.001*** |

**Table S4.** Results of Tukey HSD test for biomass of Arthrospira and Chlorella cultured under different cover treatments in time. First time step is not shown because of no variance. Cover types are shown in pairwise comparisons for each time step and divided according to algal species. Significant values for p< 0.05 are shown in bold.

|  |  | *Arthrospira* | | | |  | *Chlorella* | | | |
| --- | --- | --- | --- | --- | --- | --- | --- | --- | --- | --- |
|  |  | Film | Film 30° | Full sun | Glass |  | Film | Film 30° | Full sun | Glass |
| Time2 | Film |  |  |  |  |  |  |  |  |  |
|  | Film 30° | ***0,001*** |  |  |  |  | ***0,000*** |  |  |  |
|  | Full sun | *0,994* | ***0,001*** |  |  |  | ***0,000*** | *0,959* |  |  |
|  | Glass | ***0,004*** | *0,303* | ***0,005*** |  |  | ***0,000*** | ***0,008*** | ***0,004*** |  |
| Time3 | Film |  |  |  |  |  |  |  |  |  |
|  | Film 30° | ***0,023*** |  |  |  |  | ***0,000*** |  |  |  |
|  | Full sun | *0,996* | ***0,017*** |  |  |  | ***0,000*** | *0,746* |  |  |
|  | Glass | ***0,038*** | *0,980* | ***0,028*** |  |  | ***0,000*** | ***0,001*** | ***0,000*** |  |
| Time4 | Film |  |  |  |  |  |  |  |  |  |
|  | Film 30° | ***0,000*** |  |  |  |  | ***0,000*** |  |  |  |
|  | Full sun | *0,999* | ***0,000*** |  |  |  | *0,170* | ***0,000*** |  |  |
|  | Glass | *0,396* | ***0,000*** | *0,335* |  |  | ***0,000*** | ***0,000*** | ***0,000*** |  |
| Time5 | Film |  |  |  |  |  |  |  |  |  |
|  | Film 30° | ***0,000*** |  |  |  |  | ***0,001*** |  |  |  |
|  | Full sun | *1,000* | ***0,000*** |  |  |  | ***0,000*** | *0,082* |  |  |
|  | Glass | ***0,000*** | ***0,000*** | ***0,000*** |  |  | ***0,000*** | ***0,000*** | ***0,000*** |  |

**Table S5.** Results of MANOVA (Multivariate Analysis Of Variance) on the three species (basil, lettuce and tomato) grown in presence or absence of selective film (Light) and at different times (Time).

|  | Basil | | Lettuce | | Tomato | |
| --- | --- | --- | --- | --- | --- | --- |
|  | F | p | F | p | F | p |
| Time | 7.416 | ***0.000*** | 6.704 | ***0.000*** | 17.270 | ***0.000*** |
| Light | 41.567 | ***0.000*** | 1.908 | 0.122 | 8.239 | ***0.003*** |
| Time*Light | 6.981 | ***0.000*** | 1.415 | 0.254 | 7.328 | ***0.005*** |

**Table S6.** Dunnett’s test p-values of biometric variables of tomato, lettuce and basil plants grown under transparent glass and selective films at two time points. Significant values for p<0.05 are shown in bold.

|  | Tomato | | Lettuce | | Basil | |
| --- | --- | --- | --- | --- | --- | --- |
|  | T1 | T2 | T1 | T2 | T1 | T2 |
| Number of leaves | ***0,020*** | ***0,020*** | 0,407 | 0,109 | 0,497 | ***0,001*** |
| Shoot length | ***0,007*** | ***0,033*** | 0,612 | 0,368 | ***0,000*** | ***0,000*** |
| Root biomass | 0,508 | 0,816 | 0,367 | 0,295 | 0,166 | 0,092 |
| Shoot biomass | 0,178 | ***0,000*** | n.c. | n.c. | 0,323 | ***0,000*** |
| Leaf biomass | 0,925 | 0,198 | 0,236 | 0,945 | 0,249 | ***0,000*** |
| Total biomass | 0,469 | 0,200 | 0,087 | 0,569 | 0,630 | ***0,004*** |
| R/S | 0,421 | ***0,013*** | ***0,007*** | 0,225 | ***0,000*** | ***0,000*** |
| Total dry biomass | 0,414 | 0,796 | 0,249 | 0,277 | 0,543 | ***0,034*** |
| Root dry biomass | 0,448 | 0,260 | 0,088 | 0,198 | 0,192 | 0,266 |
| Shoot dry biomass | 0,173 | ***0,002*** | n.c. | n.c. | 0,464 | ***0,001*** |
| Leaves dry biomass | 0,856 | 0,774 | 0,706 | 0,46 | 0,529 | ***0,000*** |
